# An F2A sequence permits correct localization of a secreted and a nuclear localized reporter in *C. elegans*

**DOI:** 10.17912/micropub.biology.002351

**Published:** 2026-09-21

**Authors:** James Matthew Ragle, Guinevere Ashley, Jordan D. Ward

**Affiliations:** 1 Department of Molecular, Cell, and Developmental Biology, University of California – Santa Cruz, Santa Cruz, CA 95064, USA

## Abstract

Multicistronic expression systems enable production of multiple proteins from a single transcript, with internal ribosome entry sites (IRES), SL2 trans-splicing, and 2A peptides as common tools. Because 2A peptides rely on a single translation event, we tested whether nuclear-localized mStayGold (mSG::H2B) and secreted mScarlet (ssmScarlet) reporters separated by F2A in *C. elegans* produced the expected localization pattern. mSG::H2B::F2A::ssmScarlet and ssmScarlet::F2A::mSG::H2B expressed in body wall muscle produced nuclear mSG in muscle and ssmScarlet in coelomocytes, indicating that downstream secreted proteins could be correctly directed to the secretory pathway. These data provide a configuration for driving nuclear and secreted proteins from a single-copy transgene.

**Figure 1. An F2A sequence allows correct localization of nuclear and secreted protein reporters regardless of configuration:** Dual reporter strains of the indicated genotype were imaged for secreted mScarlet (ssmScarlet) and mStayGold::histone H2B fusions (mSG::H2B). A merged image overlaid on a DIC image is provided and the outline of the coelomocyte cell body is indicated by a dashed yellow line in the DIC overlap images. Images are representative of 20 young adult animals imaged over two independent experiments. We selected images where coelomocyte and muscle nuclei could both be imaged in a single DIC plane and animal anterior is oriented to the left. The top row contains images of the posterior pair of coelomocytes closest to the tail. The bottom three rows contain images of the mid-body pair of coelomocytes. Scale bars=10 µm.

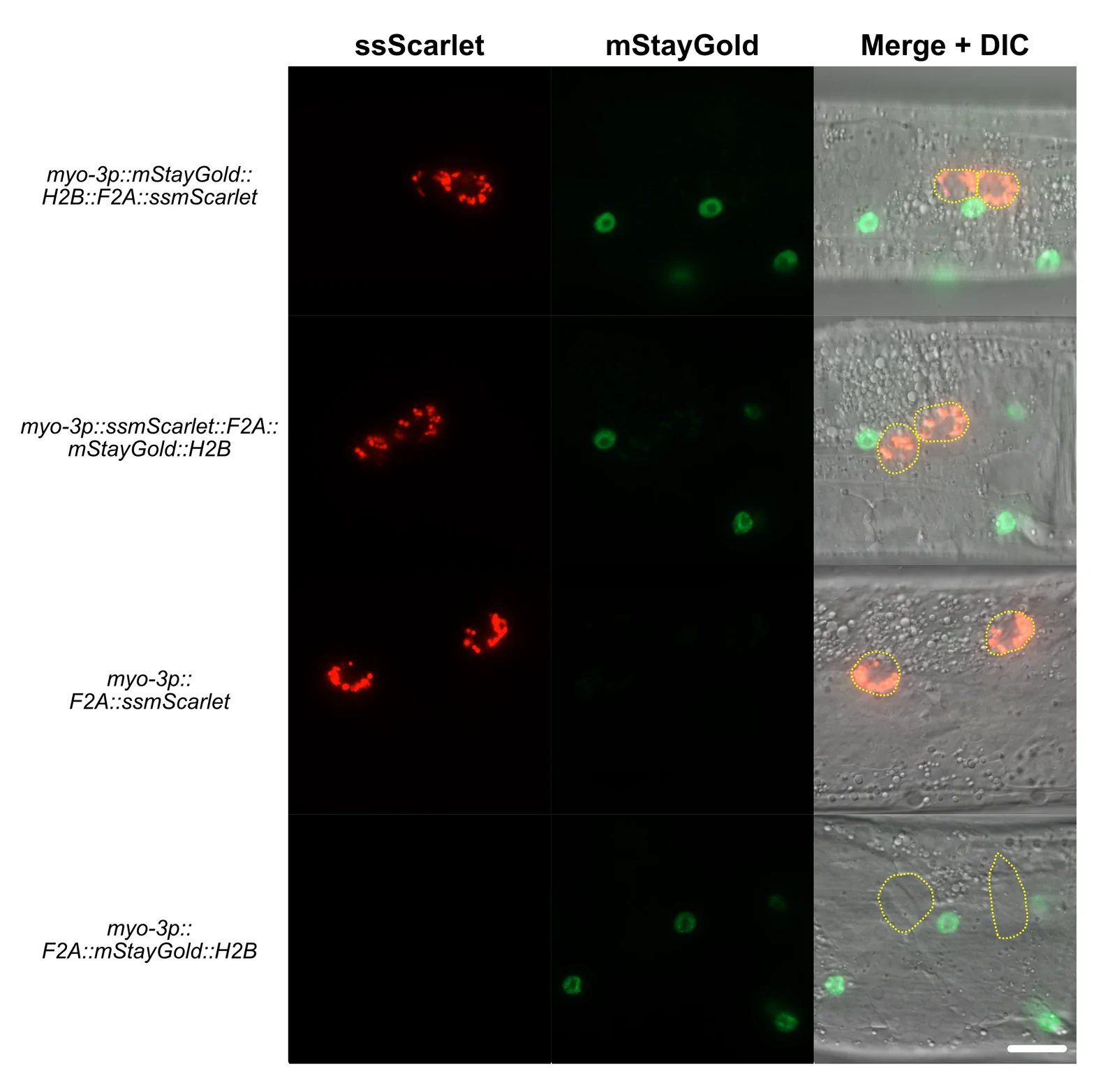



## Description

Modern transgenesis frequently involves generating multiple proteins from a single mRNA under the control of a promoter of interest. Advantages of this approach include economical packaging in targeting vectors, avoiding the need for sequential genome manipulation, and creating reporters to track the expression of unmarked proteins of interest. These sequences can also be used in genome editing to create endogenous promoter reporters, capturing all relevant *cis* -regulatory elements. Three widely used approaches are internal ribosome entry sites (IRES), SL2 trans-splicing, and 2A peptides. Internal ribosome entry sites use viral sequences that allow cap-independent initiation of translation internally within an mRNA (Martinez-Salas et al., 2017). SL2 sequences exploit polycistronic operons found in some nematode species and involve splicing an SL2 leader RNA containing the 5' cap onto a downstream gene in the operon, producing two separate monocistronic mRNAs (Blumenthal, 2005; Spieth et al., 1993). 2A peptides are viral sequences that promote a ribosomal “skipping” event during translation that yields multiple, near-stoichiometric protein products from a single open reading frame (de Lima & Lanza, 2021). IRES, SL2, and 2A sequences can also be used to tag endogenous genes, allowing reporters or other proteins of interest to be expressed under the control of a gene of interest's *cis* -regulatory elements (Nance & Frøkjær-Jensen, 2019; Wang & Marchisio, 2021).

With IRES and SL2 sequences, separate translation initiation events produce the upstream and downstream proteins (Blumenthal, 2005; Martinez-Salas et al., 2017). In contrast, with 2A sequences, a single ribosome initiates translation and peptide cleavage produces separate polypeptides (de Lima & Lanza, 2021). A proof-of-principle study established that several 2A peptides could be used in *C. elegans* to enable, from a single construct, efficient delivery of up to four proteins to distinct compartments such as the cytoplasm, nucleus, nuclear membrane or plasma membrane (Ahier & Jarriault, 2014). However, it remains unclear whether 2A sequences could simultaneously support targeting of proteins with nuclear localization signals and signal peptides. There are examples in which a cytosolic or nuclear-localized 2A::fluorescent reporter displayed correct localization downstream of a secreted protein (Rasala et al., 2012; Sun et al., 2023). However, there was a report where a downstream cytosolic 2A::reporter failed to cleave and was pulled into the secretory pathway through a proposed “slipstream” mechanism (de Felipe et al., 2010). The generalizability of this result is not clear, as another study found that a secreted protein downstream of a 2A sequence required its own signal sequence for secretion (Yan et al., 2010).

Given these variable reports and the wide use of 2A peptides in *C. elegans* transgenes (Ahier & Jarriault, 2014), we tested whether mStayGold::histone H2B (mSG::H2B) and secreted mScarlet (signal sequence mScarlet; ssmScarlet) reporters separated by an F2A sequence expressed in body wall muscle displayed the expected localization pattern (Figure 1). We chose F2A as we had used it extensively in our auxin-inducible degron work and it produced efficient cleavage (Ashley et al., 2021). Fluorescent proteins secreted from many tissues into the pseudocoelom are subsequently scavenged by coelomocytes and accumulate in these cells (Fares & Greenwald, 2001; Fitzgerald & Greenwald, 1995; Grant & Greenwald, 1997). GFP secreted from body wall muscle has been previously used to genetically dissect the endocytic pathway (Fares & Greenwald, 2001). In both configurations, we observed mScarlet signal accumulating in coelomocytes and mStayGold in muscle nuclei. Downstream mStayGold::H2B did not detectably enter the endoplasmic reticulum through a slipstream mechanism, and the ssmScarlet did appear to efficiently enter the secretory pathway after 2A cleavage. We included F2A::mSG::H2B and F2A::ssmScarlet controls lacking the upstream FP, which also localized as expected. These results provide an effective design that allows correct localization of nuclear and secreted proteins separated by an F2A sequence. This design would likely support correct localization to other cellular compartments based on efficient 2A cleavage, though this assertion will need to be tested. We note that in this study cleavage was inferred by reporter localization, and in the future western blotting experiments would be valuable to directly assess cleavage efficiency and other 2A peptides should be similarly tested. A recent study using a similar recombinase-mediated single-copy integration approach in CHO cells displayed incomplete cleavage (Ng et al., 2025). In contrast, our study supports the robust function of F2A peptides in single-copy transgenes inserted by rapid recombinase mediated cassette exchange in *C. elegans* . Together, this work indicates that expressing secreted proteins and proteins with specific sub-cellular localizations from single transgenes under promoters of interest is feasible, adding to the *C. elegans* toolkit.

## Methods

Cloning and strain generation

*F2A::mStayGold (dpi)::H2B:: ubl-1 3'UTR* (pJW2791) and *F2A::ssmScarlet (dpi):: ubl-1 3'UTR* (pJW2792) plasmids were generated by Twist Biosciences, cloning the insert into a pTwist Kan High Copy backbone. The “dpi” designation refers to sequence optimization to remove piRNA target sites performed with the pirScan program (Wu et al., 2018). These plasmids contained ATG and GTA connectors for SapTrap and a KpnI restriction enzyme site upstream of the F2A sequence to allow linearization to Gibson clone in new sequences. We amplified ssmScarlet from pJW2792 to Gibson clone into linearized pJW2791 to generate pJW2793 ( *F2A::ssmScarlet (dpi):: ubl-1 3'UTR* ). Similarly, mStayGold::H2B was amplified from pJW2791 to Gibson clone into linearized pJW2792 to make pJW2794 ( *mStayGold (dpi)::H2B::F2A::ssmScarlet (dpi):: ubl-1 3'UTR* ). pJW2791-pJW2794 were combined with pNM4104 ( *myo-3p)* into a rapid RMCE backbone (pNM4216) through SapTrap (Schwartz & Jorgensen, 2016) to generate pJW2826 ( *myo-3p::ssmScarlet (dpi)::F2A::mStayGold (dpi)::H2B:: ubl-1 3'UTR* ), pJW2827 ( *myo-3p::mStayGold (dpi)::H2B::F2A:: ssmScarlet (dpi):: ubl-1 3'UTR* ), pJW3015 ( *myo-3p::F2A::mStayGold (dpi)::H2B:: ubl-1 3'UTR* ), and pJW3016 ( *myo-3p::F2A::ssmScarlet (dpi):: ubl-1 3'UTR* ). pJW2826, pJW2827, pJW3015, and pJW3016 were integrated into NM5548 using rapid RMCE as previously described (Nonet, 2023) to generate JDW972, JDW937, JDW1041, and JDW1042, respectively. Oligonucleotides and sequence files available upon request.

Imaging

Day 1 adults were mounted on glass slides in 24 ul M9 + 0.05% gelatin and 10mM levamisole and imaged at 100 ms (Alx488) and 200 ms (Alx549) using a Plan-Apochromat 100x/1.40 Oil M27 Oil DIC lens on an AxioImager M2 microscope (Carl Zeiss Microscopy, LLC) equipped with a Colibri 7 LED light source and an Axiocam 506 mono camera. Acquired images were processed through Zen 2.3 (blue edition).

## Reagents

**Table:** 

**Plasmid**	**Reference**	**Notes**	**How to obtain plasmid**
pJW2791	This study	* F2A::mStayGold (dpi)::H2B:: ubl-1 3'UTR* for SapTrap with ATG and GTA connectors. Has a KpnI site to clone in ssmScarlet or other factors in front of F2A	Request from Jordan Ward
pJW2792	This study	* F2A::ssmScarlet (dpi):: ubl-1 3'UTR* for SapTrap with ATG and GTA connectors. Has a KpnI site to clone in ssmScarlet or other factors in front of F2A	Request from Jordan Ward
pJW2793	This study	* ssmScarlet (dpi)::F2A::mStayGold (dpi)::H2B:: ubl-1 3'UTR* for SapTrap with ATG and GTA connectors	Request from Jordan Ward
pJW2794	This study	* mStayGold (dpi)::H2B::F2A::ssmScarlet (dpi):: ubl-1 3'UTR* for SapTrap with ATG and GTA connectors	Request from Jordan Ward
pJW2826	This study	* myo-3p::ssmScarlet (dpi)::F2A::mStayGold (dpi)::H2B:: ubl-1 3'UTR* vector for rapid RMCE	Request from Jordan Ward
pJW2827	This study	* myo-3p::mStayGold (dpi)::H2B::F2A::ssmScarlet (dpi):: ubl-1 3'UTR* vector for rapid RMCE	Request from Jordan Ward
pJW3015	This study	* myo-3p::F2A::mStayGold (dpi)::H2B:: ubl-1 3'UTR* for SapTrap with ATG and GTA connectors	Request from Jordan Ward
pJW3016	This study	* myo-3p::F2A::ssmScarlet (dpi):: ubl-1 3'UTR* for SapTrap with ATG and GTA connectors	Request from Jordan Ward
pNM4104	Gift from Mike Nonet	*myo-3p* clone with TGG and ATG connectors for SapTrap	Request from Mike Nonet
pNM4216 (pHygG1)	Nonet, 2023	Insertion backbone for rapid RMCE	Request from Mike Nonet

**Table:** 

**Strain**	**Genotype**	**Available from**
NM5548	* jsSi1726 [loxP myo-2 p::FRT::nlsCyOFP:: myo-2 3' + mex-5p::FLP D5:: glh-2 3' FRT3] II *	CGC
JDW937	*jsSi1579 jsSi1706 jsSi1726 wrdSi140[loxP myo-2 p::NLS::mNeonGreen, rps-0p HygR, loxP myo-3p::mStayGold (dpi)::H2B::F2A::ssmScarlet (dpi):: ubl-1 3'UTR FRT3] II *	Prof. Jordan Ward
JDW972	* jsSi1726 wrdSi149[loxP myo-2 p::NLS::mNeonGreen, rps-0p HygR, loxP myo-3p::ssmScarlet (dpi)::F2A::mStayGold (dpi)::H2B:: ubl-1 3'UTR FRT3] II *	Prof. Jordan Ward
JDW1041	*jsSi1579 jsSi1706 jsSi1726 wrdSi175[loxP myo-2 p::NLS::mNeonGreen, rps-0p HygR, loxP myo-3p::F2A::mStayGold (dpi)::H2B:: ubl-1 3'UTR FRT3] II *	Prof. Jordan Ward
JDW1042	*jsSi1579 jsSi1706 jsSi1726 wrdSi176[loxP myo-2 p::NLS::mNeonGreen, rps-0p HygR, loxP myo-3p::F2A::ssmScarlet (dpi):: ubl-1 3'UTR FRT3] II *	Prof. Jordan Ward
